# Supplementary material for: Patterns of Intron Gain and Loss in Fungi
Source: PLoS Biol. 2004 Nov 30;2(12):e422. doi: 10.1371/journal.pbio.0020422 (PMC532390; doi:10.1371/journal.pbio.0020422)
Supplement: Table S1 — Also available at http://genes.mit.edu/NielsenEtAl/. (4.3 MB ZIP). [file pbio.0020422.st001.zip › NielsenEtAl/html/767.html]

AN7707.1.NCU06429.1.MG06475.1.FG07284.1


```
 CLUSTAL W (1.82) Multiple Sequence Alignments - Introns Inserted


Sequence 1: AN7707.1	690 aa
Sequence 2: FG07284.1	889 aa
Sequence 3: NCU06429.1	1027 aa
Sequence 4: MG06475.1	788 aa
Alignment Length: 1062 aa
Number Identitical Residues: 407 aa
Alignment Score (without introns) 18800


MG06475.1 	------------------------------------------------------------
NCU06429.1	MEMLGVEIGEPGPSVQSGQPAAALPDPATAPARAAAQSPTQSTNSTTSTRSGPASLARSR
FG07284.1 	------------------------------------------------------------
AN7707.1  	------------------------------------------------------------
          	                                                            

MG06475.1 	-----------------------------------------------------MDHQYPH
NCU06429.1	SSTAGASTTCSSRSWGETHSGGPAQVEKFPDLPTHTTSPEHQQQPLHHHPHPHHRHHHHH
FG07284.1 	-------------------------------------------MDAVRNSVQAFQGMLPL
AN7707.1  	--------------------------------------------------------MLTV
          	                                                            

MG06475.1 	DAGSVPSNREDWPLFTNENSYHP-----------------GGHTDNS-------------
NCU06429.1	QPASEAVRRDGTCAHTAEGTPTPPAAPAAPATPTPVPRLNTDHTELASPGPPP-------
FG07284.1 	QPGNLDGNEK-FPDFCLHSEALCSNDSHEDLEREQEQQHDKGLHNKQSKQRHPHFHYPRP
AN7707.1  	DESWVNVQQKTFTKWSVHPKAIPS--------------VEAGLLTR--------------
          	: .    ...       .     .               . .                  

MG06475.1 	--------------LNRPPSLSLSSTTSSYDFS------------DKDSVFDSQRASFIS
NCU06429.1	--------AASCPAIGRPPSLSIHSTASSSDHSSS--------LSDGDLVFDSRRSSVVT
FG07284.1 	HSPSASISESSRLSCISTASVSASSSTSSRVSSASFDSFSTPISPTLDSSLHSPRSSLAS
AN7707.1  	-----------------TAHVSELANVISFVLYG---------SDSADFIFYSCR-----
          	                 .. :*  :.. *    .             *  : * *     

MG06475.1 	TSSSIDSCS-------IACG-----------SPLPRSRSNAKQLALARLQRSGSNPVGLS
NCU06429.1	SASTASAYN-------YPLSPSCHQPDSSPEHPLTRTRRDAKQLHAARLERSGSISLALA
FG07284.1 	NTCSSPVSEFIARNRSLTGASLSSVSSSPPSSPTPRNRWDKPQPYAGRAQRSGSTTTNVS
AN7707.1  	------------------------------------------------------------
          	                                                            

MG06475.1 	SKKADSPS-----RKRFYSSGS---AQEALAATEPAGGGLTKMEED--WVSTQQKTFQKW
NCU06429.1	STKREAPSPALRNPTRFQRASSEPTRAPKLVTEQPAGFTKMAFAEQQQWITVQQKTFTKW
FG07284.1 	SPVGVHKQEQSSSPRSFR-SNPIPADALAAVESHDTKLIKMAFAEQQRWVTVQQKTFTKW
AN7707.1  	------------------------------------------------------------
          	                                                            

MG06475.1 	~ANSKLAERSLETKNLVEDLKDG0VLLIHLLECL0ASESLGRFASKPKLPVQQYENANTA
NCU06429.1	~LNTKIEVRGLEVKDLVKDLSDA0VMLIHLLECL~SGDSLGRYAAKPKLRVQRFENANLA
FG07284.1 	2LNTKIEARNLEVKDLVKDLSDG0VMLIHLLECL~SHESLGRYASKPKLRVQKFENANTA
AN7707.1  	~LNNKLKVRDIFVNNLVPELSNG0VTLIHLLEIL~GGDSLGRYAANPKLRVQKFENVNKS
          	  *.*:  *.: .::** :*.:. * ****** * . :****:*::*** **::**.* :

MG06475.1 	LGFIQSRGIRLTNCGAEDIVKGNRKIVL0GLIWTLILRFTISDINEEGLTAKEGLLLWCQ
NCU06429.1	LNFIKSRGIQMTNIGAEDIVDGNRKIIL~GLIWTLILRFTINDINEEGMTAKEGLLLWCQ
FG07284.1 	LDFVKSRGIQMTNIGAEDVVDGNQKIVL~GLIWTLILRFTISDINEEGMSAKEGLLLWCQ
AN7707.1  	LDYIKGRGIQMTNIGAEDIVDGNQKIIL~GLIWTLILRFTISDINEEGMTAKAGLLLWCQ
          	*.:::.***::** ****:*.**:**:* ************.******::** *******

MG06475.1 	RKTACYEECDVRDFSASWNDGLAFCALLDIHRPDLIDYDALDKTDHKGNMQMAFDIAHKE
NCU06429.1	RKTACYDEVDVRDFSGSWNDGLAFCALLDIHRPDLIDYDALDKSDHRGNMQLAFDIAHAE
FG07284.1 	RKTACYEEVEVRDFSGSWNDGLAFCALLDIHRPDLIDYDALDKADHRGNMQLAFDIAHKE
AN7707.1  	RKTACYEGVEVRDFSTSWNDGLAFCALLDIHRPDLIDYDSLDKNDHRGNMKLAFDIAANE
          	******:  :***** ***********************:*** **:***::*****  *

MG06475.1 	IGIPKLLDVEDVCDVAKPDERSLMTYIAYWFHAFSQMEKVENAGRRVEKFVNNMQGAWEM
NCU06429.1	IGIPKLLDVEDVCDVAKPDERSLMTYIAYWFHAFSQMEKVENAGRRVEKFFNNMQGAWEM
FG07284.1 	IGIPKLLDVEDVCDVAKPDERSLMTYIAYWFHAFSQMEKVENAGRRVEKFVNNMQGAWEM
AN7707.1  	VGIPDLLDVDDVCDVAKPDERSLMTYIAYWFHAFSQLERVENAGRRVEKFVNNMHGAWEM
          	:***.****:**************************:*:***********.***:*****

MG06475.1 	QSAYERRMAALLQAIRAQVESWQQAKFEGSYTDAKAQATDFASYKRGKKREWVAEKSELA
NCU06429.1	QSAYERRMAALLKAIREQVVSWKGSTFDGTYADAKAQAFQFASYKKGKKREWVAEKSDLA
FG07284.1 	QSAYERRMRALLKAIQERIEVWKEATFEGTYTDAKAQLNQFFDYKRGKKREWVAEKSDLA
AN7707.1  	QNSYEKRMRELLRLIRAQREEWKNASFEGTYKDAKEQASQFAMYKRNQKRQWVAEKSDLA
          	*.:**:**  **: *: :   *: :.*:*:* *** *  :*  **:.:**:******:**

MG06475.1 	TLLGNIKTKLGTYRLRPYEPPAELRLDVLDGEWANLAAAEMKRGQLINETIRD~IKNALR
NCU06429.1	TLLGNIKTKLATYRLRPYDPPAHLRMEVLDDEWGNLSKAEMSRGQLINETIRD2IKDALR
FG07284.1 	TLLGNIKTKLGTYRLRPYDPPAELSLDALEQRWAELASNEMRRAQLINETIRD2IKNALR
AN7707.1  	ALLGNIKTKLSTYRLRAYDPPPELSPEACDQEWECLTRDEHERSQLINETIRD~IKNALR
          	:*********.*****.*:**..*  :. : .*  *:  *  *.********* **:***

MG06475.1 	KSFADKANDFALALNTIQLAISGLEGDVEDQLHHVRKLSENLPPLDEYLKTIGAVDAKCQ
NCU06429.1	KSFADKANDFALALNTMQLAISGLEGDVEDQLHHVRKLSENLPPLDAYLKTIEAVDLKCQ
FG07284.1 	KSFADKANDFAMALNTMQLALSGLDGDVEDQLHHVRKLSESLSPLDQYLDKISELDQKCQ
AN7707.1  	RSFADKANDFALTLKTLSLAISGLDGDVEDQLAHVKRLNDNLPPLDAFLETIAELDEQCQ
          	:**********::*:*:.**:***:******* **::*.:.*.*** :*..*  :* :**

MG06475.1 	EANIEENDFTTYTYDELCYELGLVKSSVAKKLAFLDNQMVARNMTNLTPIQLEEFESVFR
NCU06429.1	EANIEENDFTTYSYDELCYELSLVKSSVSKKLAFLENQMVARNMTNLTPIQLEEFESVFR
FG07284.1 	EANIEENDFTTYTYDELSYELGLVKTSVQKKLAFLENQMVARSMTNLTPIQLEEFESVFR
AN7707.1  	EANVEENDYTTYTLDELAYELSLVKSSISKKMAFLDNQLVARNMTNLTPIQLEEFESVFR
          	***:****:***: ***.***.***:*: **:***:**:***.*****************

MG06475.1 	HFDRDDSNSLAELEFGAALASLGVVFSEGEMHEYFVETAK-----GRDRITFEQFIRFMV
NCU06429.1	HFDRDDTNSLSELEFSAALASLGLVFSEDEMHEYFLSTSN-----GRDRVTFEQFIRFMV
FG07284.1 	HFDRDDTNSLQELEFSAALASLGLVFSEDEMHDYFHATSG-----GRDYVTFEQFIRFMV
AN7707.1  	HFDRDSSNTLQELEFSAALASLGLVYDEEEMHQVYVETCGPARLAQGAGVSFEQFIRFMV
          	*****.:*:* ****.*******:*:.* ***: :  *. .:  :    ::*********

MG06475.1 	EVTEDQNTAEQVYQSFREVADGK~PYVTEMDLRHSLVPDEVIDKLIEIIPAHNGPDTAQD
NCU06429.1	DVTEDQNTAEQVFQSFREVADGK~PYVTEMDLRHSLVPDEVIEKLIEIIPKHTGPDMQSD
FG07284.1 	DVTEDQNTAEQVYQSFREVADGK~PYVTEMDLRHSLVPDEVIDQLVEIMPAHSGPDMSED
AN7707.1  	SVTEDQNTAEQVFQSFREVADGK0PYVTELDLRHSLIPDEIIEHLVKTMPQHEGPDLLED
          	.***********:********** *****:******:***:*::*:: :* * ***  .*

MG06475.1 	RGMPQYDYIAFMDKFIGSD------------------AAANGCKSPTKVTVNGH------
NCU06429.1	RGMEQYDYIAFMEKLISEQEGRAASASSRRSSRASGVAGGGGAAAPGQMPNHSHSASVGG
FG07284.1 	RGMPQYDYISFMEKLINEQNRQGGSET----------ALPDRSDPEPQSPRTNGV-----
AN7707.1  	RDLPKYDYISFMERMMNDNSSQG------------------------NAPINGSV-----
          	*.: :****:**::::..:.  .                        : .  .       

MG06475.1 	------------------------------------------------
NCU06429.1	SISHSRHNSHASIGGKSVLGGGDSRSNGLMMSPKSPGTPGTNKPNGYH
FG07284.1 	------------------------------------------------
AN7707.1  	------------------------------------------------
          	
```
